# Conformation and Membrane Topology of the N-Terminal Ectodomain of Influenza A M2 Protein

**DOI:** 10.3390/membranes15020040

**Published:** 2025-02-01

**Authors:** Kyra C. Roepke, Kathleen P. Howard

**Affiliations:** Department of Chemistry and Biochemistry, Swarthmore College, Swarthmore, PA 19081, USA

**Keywords:** influenza, M2 protein, ectodomain, spin labels, EPR, universal vaccine

## Abstract

The N-terminal ectodomain of the influenza A M2 protein is a target for universal influenza vaccine development and novel antiviral strategies. Despite the significance of this domain, it is poorly understood and most structural studies of the M2 protein have disregarded the N-terminal ectodomain in their analyses. Here, we report conformational properties and describe insights into the membrane topology of sites along the N-terminal ectodomain. Full-length M2 protein is embedded in lipid bilayer nanodiscs and studied using site-directed spin labeling electron paramagnetic resonance spectroscopy. Results are consistent with a turn in the middle of the ectodomain that changes in proximity to the membrane surface upon the addition of cholesterol or the antiviral drug rimantadine. Similarly to other domains of M2 protein, lineshape analysis suggests that the N-terminal ectodomain can adopt multiple conformations.

## 1. Introduction

Influenza is an acute viral infection that spreads easily from person to person and can affect any age group [1]. Each year, influenza epidemics strain health services and have negative economic impacts in terms of hospitalizations and work absenteeism. The genomes of influenza viruses are highly plastic and prone to mutations and interspecies transmission [2]. Consequently, new strains of influenza viruses that humans have low resistance to can emerge and cause devastating pandemics [3]. Combating influenza outbreaks is challenging. Vaccination can be one of the most effective means for preventing influenza outbreaks. However, the continuous antigenic drift of circulating viruses causes a need for yearly updates to the influenza vaccine composition and likely provides limited protection against novel pandemic strains. Antiviral drugs offer the promise of treatment [4] although resistance to antivirals has grown markedly high in some influenza strains [5]. The limitation of current vaccines and antivirals makes the development of new strategies for the prevention and treatment of influenza a worldwide healthcare priority. 

**Figure 1:**
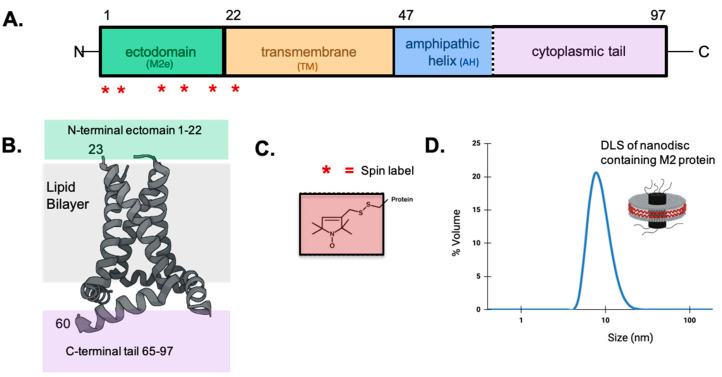
(**A**) The domain structure of the full-length 97-residue M2 protein. The location of spin-labeled sites (2, 4, 11, 14, 18, 21), indicated by red stars (*). The full sequence of M2 is included in Figure S1. (**B**) A model of a truncated homotetrameric M2 protein (residues 23–60) using previously published EPR data [11]. The work published in this paper includes ectodomain residues 1–22 not included in the ribbon model shown in 1B, but indicated by a green box. (**C**) A nitroxide spin label that is covalently bound to the sulfhydryl group of an introduced cysteine residue. (**D**) The dynamic light scattering trace of a representative M2-nanodisc sample. Stokes diameter: 11.4 ± 0.2 nm.

Fundamental information on the influenza virus life cycle and the structural properties of the viral components are still being uncovered. There has been sustained interest in matrix protein 2 (M2), which resides in the coat of the influenza virus as well as the plasma membrane of an infected cell. The M2 protein is a 97-amino-acid membrane-bound protein that functions as a homotetramer (Figure 1). The M2 protein has been studied by a wide range of biophysical techniques [6]. Each monomer consists of an N-terminal ectodomain (M2e), a transmembrane (TM) domain, and a C-terminal cytoplasmic domain containing an amphipathic helix (AH) followed by a mobile tail. The tetrameric TM is a four-helix bundle that acts as a proton channel which promotes the uncoating of virions upon entrance to a host cell. The AH plays important roles in viral budding and morphology. M2e has been linked to the successful incorporation of M2 into nascent virions [7] and has garnered significant attention as a target for universal influenza vaccines [8] and novel antiviral strategies [9,10].

The sequence of M2e is highly conserved among humans, as well as in swine and avian strains [12]. The potential for cross-species mixing, which could lead to a pandemic outbreak, makes M2e a particularly powerful target for a universal vaccine. The high conservation of M2e likely results from its genetic overlap with the open reading frame of influenza matrix protein 1 [8]. Monoclonal antibodies against M2e have been developed, and their therapeutic potential has been explored. Several M2e-based vaccines are undergoing clinical trials [12].

In this paper, we provide insights into the properties of M2e using site-directed spin labeling electron paramagnetic resonance (SDSL-EPR). SDSL-EPR is a technique that is highly suitable for detecting the mobility and membrane topology of membrane proteins reconstituted into lipid bilayers [13]. SDSL-EPR has been previously used to study the C-terminal extramembranous domain of full-length M2 protein [14,15,16,17], which provides valuable context for the interpretation of M2e data.

Partial models for the conformation of M2e have been proposed [18]. Two X-ray structures of short M2e peptides bound to protective monoclonal antibodies have been published [18,19]. The folds of the M2e peptides differ in the two complexes, but both include a turn. A solid-state nuclear magnetic resonance model (ssNMR) suggested beta-strand character within M2e based on chemical shift patterns [20]. Another ssNMR study proposed that M2e is unstructured and dynamic [21]. The observation that M2e appears unstructured under some conditions, yet can form secondary structure motifs in other conditions, has been hypothesized to result from M2e’s ability to shift between different unique conformations [19]. In this paper, we designed our experiments to avoid artifacts that could result from using a truncated protein or excluding a membrane surface. Thus, we measured conformational properties of M2e within a full-length M2 protein embedded in lipid bilayers. We also investigated how M2e was impacted upon the addition of each of two molecules (cholesterol and an antiviral drug) previously shown to modify the properties of the other domains of M2.

## 2. Materials and Methods

### 2.1. Expression, Spin Labeling, and Purification of Spin-Labeled Full-Length M2 Protein

Single cysteine substitutions were introduced into a cysteine-less background plasmid based on the A/Udorn/72 M2 sequence [22] using site-directed mutagenesis (Sequences are shown in Figure S1). To minimize potential perturbations caused by the replacement of a residue with a nitroxide spin label, we used only one label at a time, chose a spin label that is similar in size to a natural amino acid and investigated multiple proximal sites within the M2e domain to provide a valuable internal comparison of the measured values. A large number of studies on M2 have shown that the introduction of a single cysteine outside the TM core leads to only minor changes in function in the majority of cases [23,24].

Full-length M2 protein was over-expressed and purified using methods previously optimized in our laboratory [16]. Briefly, a C-terminal His-tagged protein was expressed in *E. coli* cells and purified using nickel affinity chromatography. A spin label was covalently linked via a disulfide linkage to the introduced cysteines with the S-(1-oxyl-2,2,5,5-tetramethyl-2,5-dihydro-1H-pyrrol-3-yl) methyl methanesulfonate (MTSL) spin label (Sigma).

### 2.2. Reconstitution of Spin-Labeled M2 Protein into Nanodiscs

Full-length M2 protein was reconstituted into nanodiscs using a protocol optimized in our lab [14]. Briefly, we assembled nanodiscs with a membrane bilayer consisting of 1-palmitoyl-2-oleoyl-sn-glycero-3-phosphocholine (POPC, Avanti Polar Lipids, Alabaster AL): 1-palmitoyl-2-oleoyl-sn-glycero-3-phosphoglycerol (POPG, Avanti Polar Lipids, Alabaster AL) in a molar ratio of 4:1. Lipid compositions with a range of complexities and similar to a physiological viral lipid composition have been used in prior biophysical studies [16]. The lipid mix we chose for this study has been demonstrated to allow for budding, which M2 facilitates in the plasma membrane [25]. Previous M2 SDSL-EPR studies [14,15,16,17] have used this POPC:POPG 4:1 lipid mix, comprising a body of data to with which to compare our results. Native mass spectrometry demonstrated that M2-nanodiscs composed of bilayered lipids with a similar hydrophobic thickness to that of our lipids selectively contained tetrameters [26]. Based on the mean surface area needed to accommodate tetrameric M2 protein [27], we used the membrane scaffold protein construct MSP1D1. The molar ratio of M2:MSP1D1:lipids was 4:2:130 [14]. The mixture was incubated for 2 h at 4 °C. An amount of 1.5 g of Biobeads (Bio-Rad, Hercules, CA) was mixed with 20 mM Tris pH 7.8 and 100 mM NaCl, and degassed. The Biobeads were added in 3 equal aliquots, the first 2 aliquots being added 2 hrs apart and the 3^rd^ being incubated for an additional 12 h. After Biobead removal, the nanodisc solution was passed through a 0.45 μm filter and characterized using dynamic light scattering.

### 2.3. Composition of Cholesterol and Drug Samples

Cholesterol-containing lipid films were prepared by adding a 30% molar fraction of cholesterol (Sigma) to 4:1 POPC:POPG lipid preparations, in accordance with previous literature [16]. Nanodiscs were then prepared identically to non-cholesterol-containing samples.

The protocol for the incorporation of the rimantadine drug into M2 embedded in lipid bilayers was described in a previous publication [28]. Briefly, the rimantadine drug was dissolved in trifluoroethanol (TFE) at 5 mg/mL. Aliquots of the solution were added to a conical glass vial, and TFE was removed under a gentle stream of nitrogen gas followed by placing the vials under high-vacuum conditions for ~24 hrs to remove the residual solvent. Prepared M2-nanodisc samples were incubated with rimantadine film for 24–48 h, which was followed by extensive mixing. The samples had a 1:20 molar ratio of M2 tetramer to rimantadine.

### 2.4. Dynamic Light Scattering

Dynamic light scattering (DLS) was used to measure the size of the nanodiscs to confirm proper nanodisc formation. Nanodisc samples were spun at 14,000 rpm in a benchtop microfuge for 10 min to remove any large particulates prior to DLS measurement. DLS was collected on Malvern Zetasizer Nano-ZS using 1 cm pathlength cuvettes. Data were reported as an average of five measurements.

### 2.5. EPR Spectroscopy and Data Analysis

Continuous-wave (CW) and power saturation spectra were collected on an X-band Bruker EMX spectrometer equipped with an ER4123S resonator at room temperature. CW spectra were acquired in gas-permeable TPX capillary tubes using a 2 mW incident microwave power, a 1 G field modulation amplitude at 100 kHz, and a 150 G sweep width. The number of scans varied in accordance with signal quality, ranging from 3 to 9 scans. The dynamics of a spin-labeled site were assessed from the CW EPR line shape using a semi-empirical mobility factor [13] calculated from the inverse peak-to-peak width of the central line (ΔH^−1^). For power saturation spectra, experiments were performed under nitrogen gas or equilibrated with ambient air. EPR spectra were collected over 8 power levels for nitrogen power saturation experiments and 16 power levels for oxygen power saturation. The number of scans varied in accordance with signal quality, which was determined during CW spectra collection. Data were fitted to obtain ΔP_1/2_ parameters, as described previously, and errors were reported as 95% confidence intervals from the fits to the power saturation curves [11].

## 3. Results

To investigate the conformational properties and membrane topology of M2e, we generated six different constructs of the full-length M2 protein, each with a single spin label spread out along the 23-residue domain (Sequences are shown in Figure S1). Five out of the six sites we looked at (L4, I11, E14, R18 and D21) were previously site-specifically studied in a ssNMR study on M2e [21], which provides valuable results to compare our results with. To minimize the potential perturbation caused by the replacement of a residue with a nitroxide spin label, we used only one label at a time, chose a spin label that is similar in size to a natural amino acid and investigated multiple proximal sites within the M2e domain to provide a valuable internal comparison. We have previously sequentially placed nitroxide spin labels at over 25 sites in the other domains of the M2 protein [11,14,15,16,17,28].

In this paper, the spin-labeled proteins were reconstituted into lipid bilayer nanodiscs, as described in the Section 2.2. As presented in detail below, EPR data on the mobility and accessibility of paramagnetic relaxation agents of sites along M2e are used to build conformational models.

### 3.1. Dynamic Properties of Sites Along the Ectodomain

**Figure 2:**
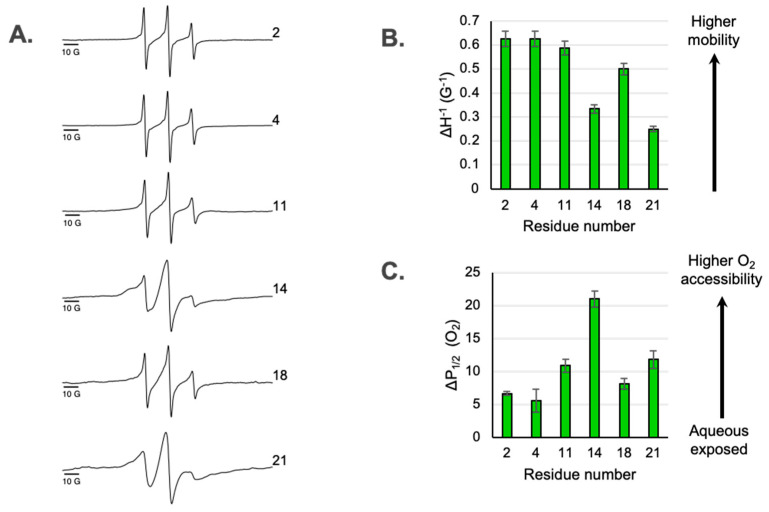
(**A**) X-band CW-EPR line shapes of spin-labeled M2 reconstituted into nanodiscs. Locations of sites within M2e are shown in Figure 1. (**B**) Mobility factors (ΔH^−1^) as a function of spin label position. The mobility factors were calculated as the inverse linewidth of the central peak from the CW-EPR spectra shown in part A. Error bars represent the uncertainty in the position of the peak maxima and minima. (**C**) Access to oxygen measured by power saturation EPR as a function of spin label position. Error bars represent the 95% confidence intervals from the fits to the power saturation curves. P_1/2_ (O_2_) values are reported in Table S1.

The EPR line shapes of spin-labeled residues on a membrane protein can provide insights into conformational dynamics and secondary structure [13]. The X-band CW EPR line shapes for the six spin labeled sites located in M2e are shown in Figure 2A. The dynamics of a spin-labeled site can be assessed from the line shape using a semi-empirical mobility factor [13] calculated from the inverse peak-to-peak width of the central line (ΔH^−1^) (Figure 2B). If the ectodomain is disordered and simply extends from the membrane surface, one can expect that the mobility will smoothly increase, moving from the sites closest to the TM domain toward the N-terminal tail. However, we observe a deviation from that predicted pattern. While site 21 closest to the TM domain is substantially immobilized compared to site 2 near the end of N-terminal tail, site 14 located near the middle of the ectodomain is immobilized compared to the sites on either side of it. 

The line shapes of the two most immobilized sites in M2e (site 14 and 21) exhibit a multicomponent nature (Figure 2). The superposition of a broad, immobilized component and a sharper, mobile component is most pronounced for site 14. Expanded spectra highlighting the multicomponent nature of line shapes are shown in Figure S2. 

The C-terminal AH was previously shown to have multicomponent EPR spectra, which were extensively characterized using both CW EPR line shape analysis and pulsed EPR saturation recovery methods [16]. This work, along with a range of complementary biophysical methods from different groups, established the presence of an equilibrium involving at least two different conformational substates of the C-terminal AH.

### 3.2. Access to Paramagnetic Relaxation Agents Along the Ectodomain

Power saturation experiments have been widely used in the SDSL-EPR of the membrane protein to determine a residue’s accessibility to paramagnetic reagents such as hydrophobic O_2_ and water-soluble paramagnetic reagents (nickel(II) ethylenediaminediacetate, NiEDDA) [13]. O_2_ preferentially partitions into lipid bilayers and NiEDDA is a hydrophilic compound that remains in the aqueous phase. Previously, our lab integrated O_2_ collision frequencies with the collision frequency of NiEDDA for both the amphipathic helix [11] and the cytoplasmic tail [15], and calculated an empirical contrast parameter, ϕ, that combines the accessibilities of O_2_ and NiEDDA. For example, analysis of spin labels placed along sites 50–60 in M2 showed a sinusoidal variation in oxygen accessibility with a periodicity of 3.6, characteristic of a surface-associated alpha helix. We noted that the NiEDDA variation along the AH (the extramembranous region on the opposite side of the membrane from M2e) is much smaller than the corresponding O_2_ variation, which often fell within error of the experiment [11]. We therefore prioritized O_2_ power saturation data in this first report on M2e.

If the ectodomain linearly extends from the membrane surface and O_2_ accessibility simply reflects membrane depth, one can expect that O_2_ accessibility would smoothly decrease as one moves from the sites closest to the TM out to the N-terminus. However, we observed a deviation from this predicted pattern at site 14. While site 21 closest to the TM shows higher oxygen accessibility than site 2 near the N-terminal tail, site 14 deviates from this pattern and has more accessible O_2_ than sites on either side of it. One possible explanation for this is that site 14 is more exposed to the hydrophobic membrane than nearby sites, which is consistent with site 14 being part of a membrane-embedded turn.

Derivatives of bilayer-forming lipids with spin labels attached at different carbon atom positions on the hydrocarbon chains have been used in the past as depth gauges for membrane bilayers [29], but determining quantitative distances near the membrane surface is complicated by a lack of distance calibration points near and above the glycerol backbone [30]. Depth gauges for reference points within the hydrophobic interior of nanodiscs with the sample composition used in this paper have been reported [14]. The P_1/2_ (O_2_) values for two depth gauges are shown in Table S1 and act as reference points for the values we report here. 

### 3.3. Impact of Cholesterol and Drug on Properties of M2e

**Figure 3:**
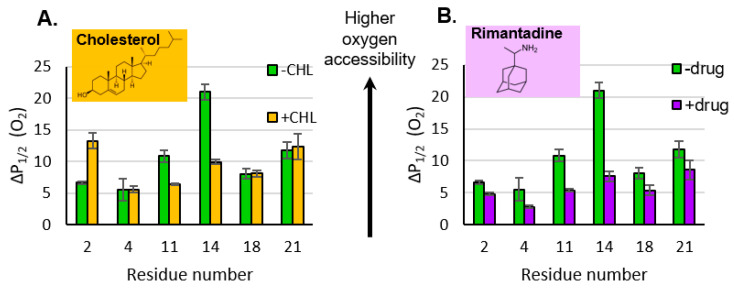
Oxygen accessibility of sites in M2e in the presence of cholesterol ((**A**) orange bars) and the drug rimantadine ((**B**) purple bars). Green bars represent samples that do not include either cholesterol or the drug. Access to oxygen is measured by the power saturation EPR as a function of the spin label position. Error bars represent the 95% confidence intervals from the fits to the power saturation curves. The ΔP_1/2_ (O_2_) values used to create the bar graphs are reported in Table S1.

Upon the addition of cholesterol and the drug, there are only minor changes in M2e CW-line shapes (Figure S2). The mobility factors calculated from the line shapes do not change within error. However, M2e sites do exhibit marked changes in oxygen accessibility (Figure 3) upon the addition of cholesterol and the drug

The addition of cholesterol can decrease the oxygen permeability of membrane bilayers [31] which complicates the interpretation of cholesterol-induced changes to membrane protein structure. In our previous work, with the same lipid composition as that used in this study, we saw an ~30% decrease in oxygen accessibility for lipid-based depth gauges [16]. Intriguingly, the changes in O_2_ accessibility for M2e site 14 is larger than expected with regard to the impact of cholesterol on O_2_ permeability; sites 4 and 18 do not show an impact of cholesterol at all and site 2 shows an increase in O_2_ (Figure 3A). This pattern of changes in O_2_ accessibility suggest that more is going on than just cholesterol-induced oxygen permeability, and changes in secondary structure or proximity to the membrane surface may perhaps have an influence on the observed data. 

Upon the addition of the drug, P_1/2_ (O_2_) for site 11 decreases by a factor of ~2 and that for site 14 decreases by a factor of ~3 (Figure 3B). These changes are consistent with the center of the M2e domain moving away from the membrane surface. The biologically relevant binding site for the rimantadine drug is located within the N-terminal half of the TM channel [6], and a range of biophysical strategies have demonstrated drug-induced conformational and dynamic changes in the protein [32].

## 4. Discussion

The mobility and oxygen accessibility results reported above provide a rich source of data with which to build models for M2e. As described below, the current understanding of the other domains of the M2 protein provide a valuable context in which to interpret the novel results we report here for M2e.

### 4.1. Comparison of the Two Extramembranous Domains of M2

**Figure 4:**
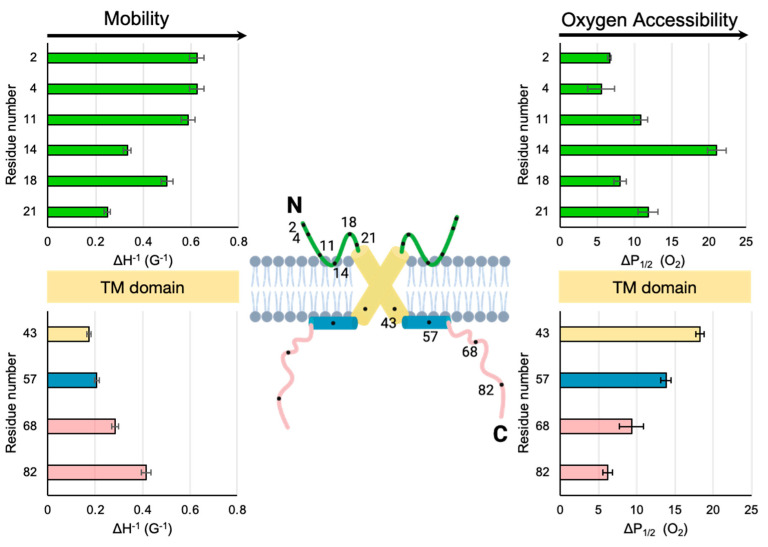
Comparison of mobility and membrane depths for the two extramembranous domains of M2. For clarity, the cartoon only shows two of the four subunits of the M2 homotetramer. The illustrated model contains values consistent with the N-terminal data reported here as well as previously published data (site 43 near the end of the TM domain, site 57 in the middle of the AH and sites 68 and 82 within the cytoplasmic tail) for the C-terminal domain of full-length M2 protein embedded in nanodiscs with the same sample composition as that used in this study [14]. Green = M2e. Yellow = TM. Blue = C-terminal AH. Pink = C-terminal tail.

It is useful to compare the SDSL-EPR data reported here for M2e to those of the more extensively studied C-terminal domain of M2. The mobility factors and O_2_ accessibility factors of the two extramembranous M2 domains are shown side by side in Figure 4. The 50-residue C-terminal domain consists of a proximal AH that lies on the membrane surface [17] followed by a distal cytoplasmic tail that dynamically extends from the membrane [15]. In terms of mobility, the least dynamic site (smallest ΔH^−1^) for both the N-terminal domain (site 21) and C-terminal domain (site 43) is the site closest to the membrane-embedded TM. Conversely, the sites closest to both the N-termini (sites 2 and 4) and C-termini (site 82) are highly dynamic. In addition, we can compare the oxygen accessibilities in M2e with previously published values for C-terminal AH of the M2 protein. Note that M2e site 14 is more oxygen-accessible than the hydrophobic face of the amphipathic C-terminal helix (site 57). The oxygen accessibility of the site closest to the N-terminus (site 2) is similar to that measured for the site closest to the C-terminus (site 82).

For the C-terminal domain, the trends in mobility and accessibility are inversely related, which has been attributed to membrane binding being a primary determinant of the reduced motion of a site [15]. For the N-terminal domain, the pattern of mobility also mirrors that of oxygen accessibility. For example, site 14 has low mobility and high oxygen accessibility. This observation is consistent with an immobilized membrane-embedded turn (Figure 4).

The TM and C-terminal domains of the M2 protein have been previously demonstrated to have multiple low-energy conformational states [16,33,34]. Thus, it is not surprising that M2e would have multiple conformations, as suggested by the multicomponent line shapes seen in Figure 2A and Figure S2. Plasticity in the packing of the four helices in the TM could propagate out to the N-terminal and C-terminal extramembranous domains. The loop regions that connect the TM to the extramembranous domains could act as flexible hinges that allow for different packing arrangements and membrane depths. The M2 protein has at least two functions: the proton channel activity crucial for uncoating of virions [35] and the generation of curvature of the plasma membranes of infected cells essential for viral budding [25]. The virion and plasma membrane have different compositions, and the properties of M2 are very sensitive to the membrane composition [16].

Our hypothesis that M2e samples multiple conformations is consistent with previously published work which showed the potential of the ectodomain to have different folds, yet appear disordered in other studies [18–19, 21]. It is unclear how a highly flexible M2e protein could serve as an antigen for high-affinity M2e-specific antibodies, but theories have been proposed in the literature [8]. The details of how M2e vaccines operate are still being determined. M2e-specific antibodies show very low binding to the surface of virions. Instead, the protective effect of anti-M2e antibodies likely results from their binding to M2e on the surface of infected cells, which triggers the removal of infected cells. Thus, high-resolution details of the conformation of the ectodomain of M2 protein embedded in a lipid membrane that mimics the plasma membrane could be valuable in elucidating the atomic-level interactions of the M2 protein with the immune system.

### 4.2. Putative Membrane Insertion Motif Within the Ectodomain

Previously published work provides clues for why M2e might include a membrane-embedded turn. Arg/Trp pairs are a well-known motif for the interaction of peptides with membrane surfaces [36]. For example, antimicrobial peptides which rely on membrane interaction for their function are often Trp- and Arg-rich and contain a well-defined turn between the two residues [36]. M2e contains a highly conserved Arg/Trp pair in the middle of the domain (Arg12 and Trp15).

Aromatic residues are capable of strong cation-π interactions with Arg residues. A cation-π interaction with an aromatic residue makes the insertion of a charged Arg residue into the hydrophobic membrane energetically more favorable. Once the insertion of an Arg into the membrane surface is facilitated by a cation-π interaction with an aromatic residue, Arg can hydrogen-bond with other species at the lipid interface. We hypothesize that a cation-π interaction promotes the formation of a membrane-embedded turn in M2e (Figure 4) that could lead to high O_2_ accessibility and low mobility near site 14.

### 4.3. Cholesterol and the Ectodomain

M2 is known to cluster in cholesterol-rich lipid rafts during viral budding from the host cell [37,38] and there has been extensive interest in the impact of cholesterol on the conformational properties of M2 [39,40]. The fact that cholesterol can impact the intrinsic oxygen permeability of a membrane bilayer complicates the interpretation of the accessibility data we report in this paper. However, previously published work has shown that cholesterol reduces the membrane depth of the C-terminal AH, which was hypothesized to result from a condensing effect on the lipid bilayer that decreases the ability of the membrane-associated AH to penetrate into the membrane [16]. The same movement away from the membrane surface upon the addition of cholesterol could be happening for M2e (Figure S3). Alternatively, cholesterol-induced changes in the TM that result from direct cholesterol binding or constrained TM movement in the rigidified bilayer have been hypothesized to lead to changes in the C-terminal domain through interdomain coupling [16]. The changes that cholesterol induces in the TM could be propagated out to M2e and lead to a change in membrane proximity.

### 4.4. Drug Binding Impacts Ectodomain Properties

As shown in Figure 3, drug binding reduces the O_2_ accessibility of all sites on M2e, with the most marked changes occurring near site 14 in the middle of the domain. This is consistent with M2e moving away from the membrane surface upon drug binding (Figure S3).

Adamantane drugs (including rimantadine, which was used in this study) contain a hydrophobic adamantane group and a positively charged ammonium group. These drugs have represented widely used models for influenza antiviral development, although more than 90% of circulating influenza A strains are currently resistant [41]. The biologically relevant binding site for rimantadine is located within the N-terminal half of the TM channel, with the large hydrophobic adamantane group located near Val27 and Ala30 and the positively charged ammonium group pointing towards the His37 residues [6].

Electrophysiological experiments have shown that an ectodomain-truncated M2 construct has weaker drug affinity than a full-length M2 protein [42] which one study attributes to the ectodomain modulating the conformation and dynamics of the TM [21]. Adamantane drug binding has been shown to narrow the wild-type TM channel and reduce its motion [32]. The impact of drug binding on the C-terminal AH has been explored by both ssNMR [40] and SDSL-EPR [28]. The C-terminal AHs are more closely packed in the presence of drugs, likely due to being tethered to the drug-immobilized TM [28]. In addition, most membrane-embedded sites on the C-terminal AH move away from the membrane upon the addition of adamantane drugs. This is consistent with M2e moving away from the membrane surface upon drug binding (Figure S3).

## 5. Conclusions

In the face of high levels of antiviral drug resistance and constantly evolving influenza strains, the high level of conservation of M2e makes it a promising target for a universal influenza vaccine and antiviral drug. A key step toward advances in disease prevention and treatment is further elucidating the structure of M2e. To best mimic the native environments of M2 and maximize the biological relevance of our work, we used a full-length M2 protein embedded in lipid bilayer membranes. We studied multiple sites along the length of M2e and measured both mobility factors and O_2_ accessibility in order to elucidate the structure and dynamics of M2e. Our data are consistent with the presence of a membrane-embedded turn in the middle of the ectodomain potentially facilitated by a cation-π interaction between Arg12 and the nearby aromatic residue at site 15. The addition of cholesterol and of the antiviral drug rimantadine changed the O_2_ accessibility of M2e, especially near the proposed membrane-embedded turn. Growing interest in M2e-targeting nanobody drugs highlights the need for an in-depth understanding of the structure and dynamics of M2e in different biologically and medically relevant environments [10]. The data reported here provide a valuable resource for developing antiviral drugs and vaccines, and may allow for improved and targeted methods for generating M2e antibodies.

## Data Availability

The original contributions presented in this study are included in the article/Supplementary Material. Further inquiries can be directed to the corresponding author.

## Supplementary Materials

The following supporting information can be downloaded at https://www.mdpi.com/article/10.3390/membranes15020040/s1: Figure S1. Sequences of the six different constructs of the full-length M2 protein. Figure S2. Overlay of X-band CW EPR line of spin-labeled M2 reconstituted into nanodiscs in the presence of cholesterol and the drug rimantadine. Figure S3. Proposed models for M2e in the presence of cholesterol and the drug rimantadine. Table S1. ΔP_1/2_ values for spin labels on M2 protein embedded in nanodiscs.
