# Supplementary material for: Conformation and Membrane Topology of the N-Terminal Ectodomain of Influenza A M2 Protein
Source: Membranes (Basel). 2025 Feb 1;15(2):40. doi: 10.3390/membranes15020040 (PMC11857740; doi:10.3390/membranes15020040)
Supplement: Supplementary file 1 [file membranes-15-00040-s001.zip › membranes-3419376-supplementary.pdf]

Supplementary Materials

Conformation and Membrane Topology of the N-Terminal Ectodomain of Influenza A M2 Protein

Kyra Roepke and Kathleen P. Howard *
Department of Chemistry and Biochemistry, Swarthmore College, Swarthmore, PA 19081, USA

* Correspondence: khoward1@swarthmore.edu

Supplementary material.

Figure S1. Sequences of the six different constructs of the full-length M2 protein.
Figure S2. Overlay of X-band CW EPR line of spin-labeled M2 reconstituted into nanodiscs in the presence of cholesterol and rimantadine drug.
Figure S3. Models that incorporate the mobility and O_2 accessibility data for M2e in the presence of cholesterol and rimantadine drug.
Table S1. $\Delta P_{1/2}$ values for spin-labels on M2 protein embedded in nanodiscs.

Figure SI. Sequences of the six different constructs of the full-length M2 protein used in this paper. Residues 22-97 were identical to wildtype for all constructs. Each has a single cysteine (red) that is used to attach a spin label. Green = M2e. Yellow = TM. Blue = C-terminal amphipathic helix. Pink: C-terminal tail.

A. **MSLLTEVETPIRNEFGSRSNDSSDPLVVAASIIGILHLILWILDRLFFKSIYRFFEHGLKRGPSTEGVPE**
MREEYRKEQQSAVDADDSHFVSIEGR

B.

```
L4   MSLCTEVETPIRNEFGSRSNDS . . .
S2   MCLLTEVETPIRNEFGSRSNDS . . .
I11  MSLLTEVETPCRNEFGSRSNDS . . .
E14  MSLLTEVETPIRNCFGSRSNDS . . .
R18  MSLLTEVETPIRNEFGSCSNDS . . .
D21  MSLLTEVETPIRNEFGSRSNCS . . .
```

Figure S2. Overlay of X-band CW EPR line of spin-labeled M2 reconstituted into 4:1 POPC:POPG nanodiscs in the presence of cholesterol and rimantadine drug. Dark gray shading highlights mobile (m) peak components. Light gray shading highlights immobile (i) peak components.

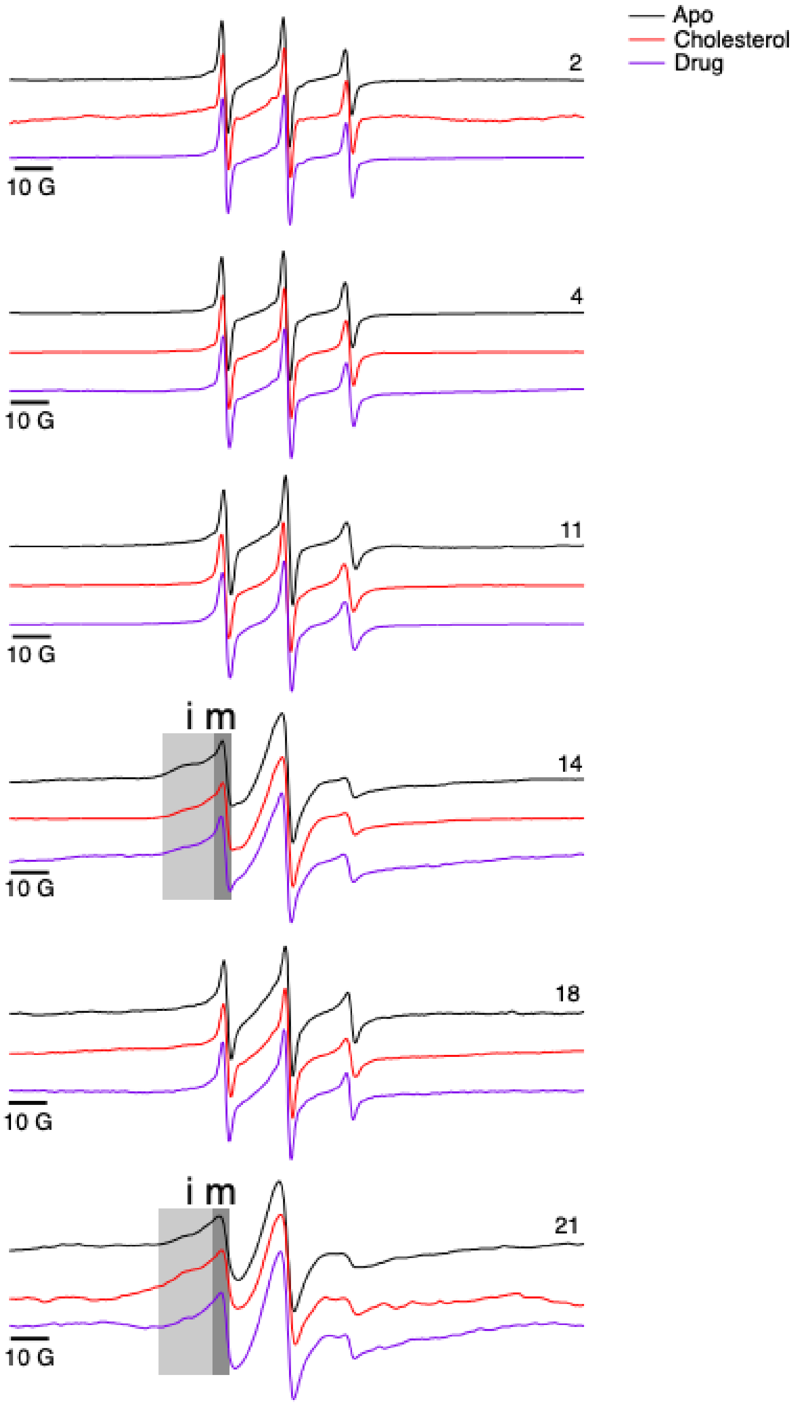

Figure S3. Models that incorporate the mobility and O_2 accessibility data for M2e in the presence of cholesterol and rimantadine drug. Green = M2e. Yellow = TM. Blue = C-terminal amphipathic helix. Pink: C-terminal tail. Purple hexagon = rimantadine drug. Cholesterol is shown as yellow/orange molecules embedded in the lipid bilayer.

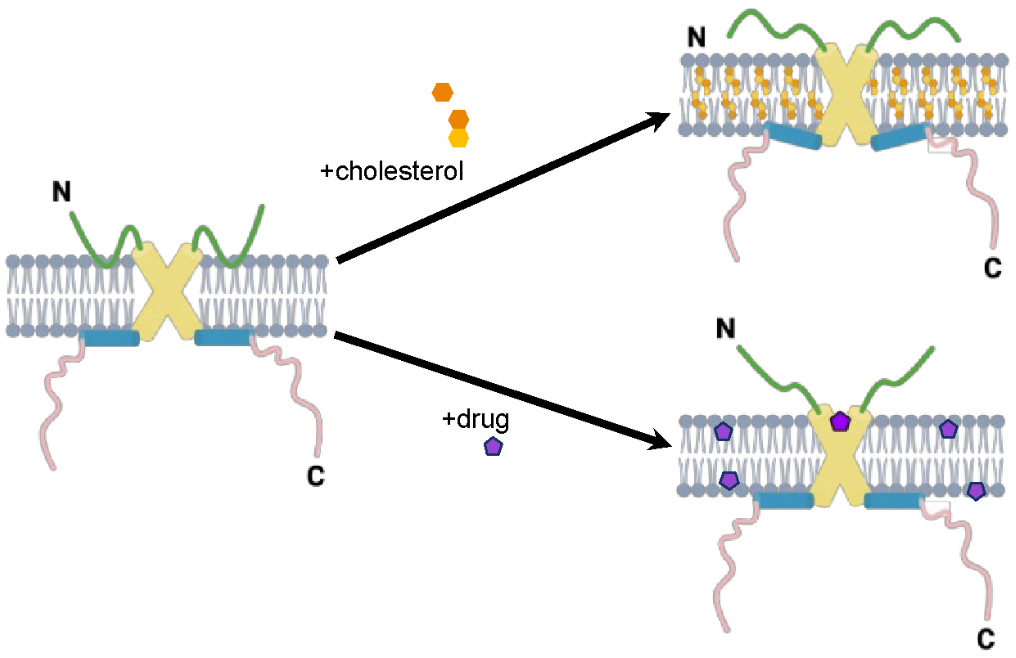

Table S1. Accessibility data for spin-labels on M2 protein and DOXYL-lipids embedded in nanodiscs. M2 data is shown in graphical form in Figures 2-4 in the main text. DOXYL-lipid data from nanodiscs with the same composition was previously published (Kyaw, 2023). Accessibility to oxygen measured by power saturation EPR as a function of spin label position. Errors reported are the 95 % confidence intervals from the fits to the power saturation curves.

**Spin-label site on M2 full-length protein**	$\Delta P_{1/2}$ (O_2) in standard condition	$\Delta P_{1/2}$ (O_2) in the presence of cholesterol	$\Delta P_{1/2}$ (O_2) in the presence of rimantadine drug
2	6.6 ± 0.2	13.3 ± 1.2	4.8 ± 0.2
4	5.6 ± 1.8	5.6 ± 0.5	2.8 ± 0.2
11	10.8 ± 0.9	6.4 ± 0.2	5.4 ± 0.3
14	21.0 ± 1.2	9.9 ± 0.3	7.6 ± 0.8
18	8.1 ± 0.8	8.1 ± 0.5	5.4 ± 0.8
21	11.8 ± 1.3	12.3 ± 2.1	8.6 ± 1.5
**X-DOXYL-labeled lipid**			
5-DOXYL lipid	15 ± 1		
10-DOXYL lipid	20.4 ± 5		
